# Supplementary material for: Single cell sequencing reveals that CD39 inhibition mediates changes to the tumor microenvironment
Source: Nat Commun. 2022 Nov 8;13:6740. doi: 10.1038/s41467-022-34495-z (PMC9643495; doi:10.1038/s41467-022-34495-z)
Supplement: Supplementary file 1 — Supplementary Information [file 41467_2022_34495_MOESM1_ESM.pdf]

Supplementary Information files

Single cell sequencing reveals that CD39 inhibition mediates changes to the tumor microenvironment in bladder cancer

Lilong Liu[1*], Yaxin Hou[2*], Changqi Deng[1], Zhen Tao[3✉], Zhaohui Chen[1✉], Junyi Hu[2✉], Ke Chen[2✉]

[1]Department of Urology, Union Hospital, Tongji Medical College, Huazhong University of Science and Technology, Wuhan, China.

[2]Department of Urology, Tongji Hospital, Tongji Medical College, Huazhong University of Science and Technology, Wuhan, China.

[3]Department of Radiation Oncology and Cyberknife Center, Tianjin Medical University Cancer institute & Hospital, Tianjin, China.

[*]These authors contributed equally: Lilong Liu and Yaxin Hou.

[✉]email: ztao@tmu.edu.cn (Zhen Tao); zhaohuichen@hust.edu.cn (Zhaohui Chen); 531572782@qq.com (Junyi Hu); shenke@hust.edu.cn (Ke Chen)

Supplementary Figure 1

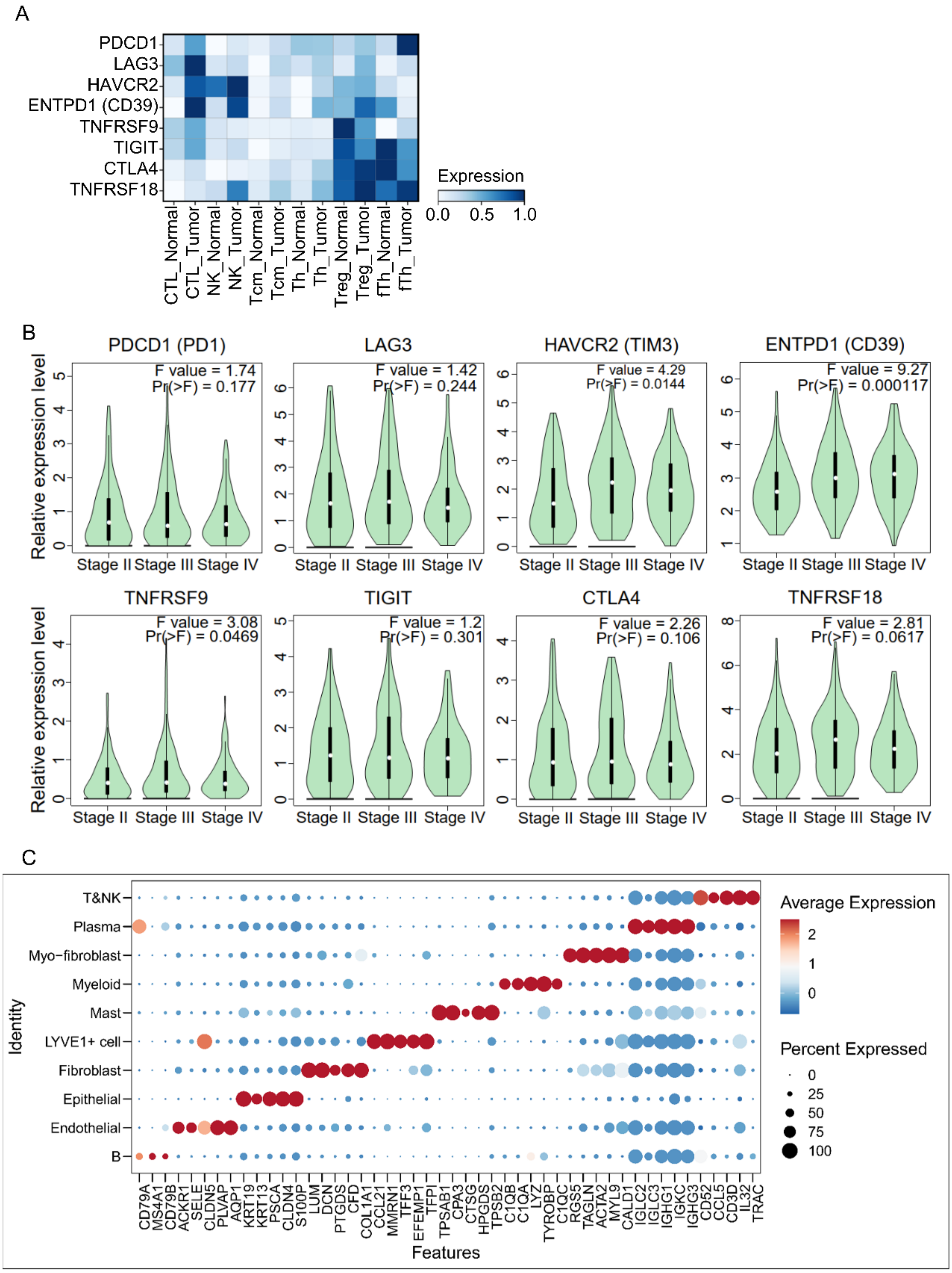

Supplementary Figure 1. The expression of CD39 in BC increased and correlated with the BC progression. A. By analysing our previously published data (8 tumor samples and 3 paratumor samples from BC patients)[1], numerous immune checkpoints were found to be prominently increased in tumor-derived lymphocytes. B. Analysis of TCGA-BLCA data using GEPIA[2] suggested that CD39 was significantly associated

with BC progression (Stage II: n=131, Stage III: n=141, Stage IV: n=136). C. Known markers used in clustering analysis. *P* values < 0.05 were considered significant.

Supplementary Figure 2

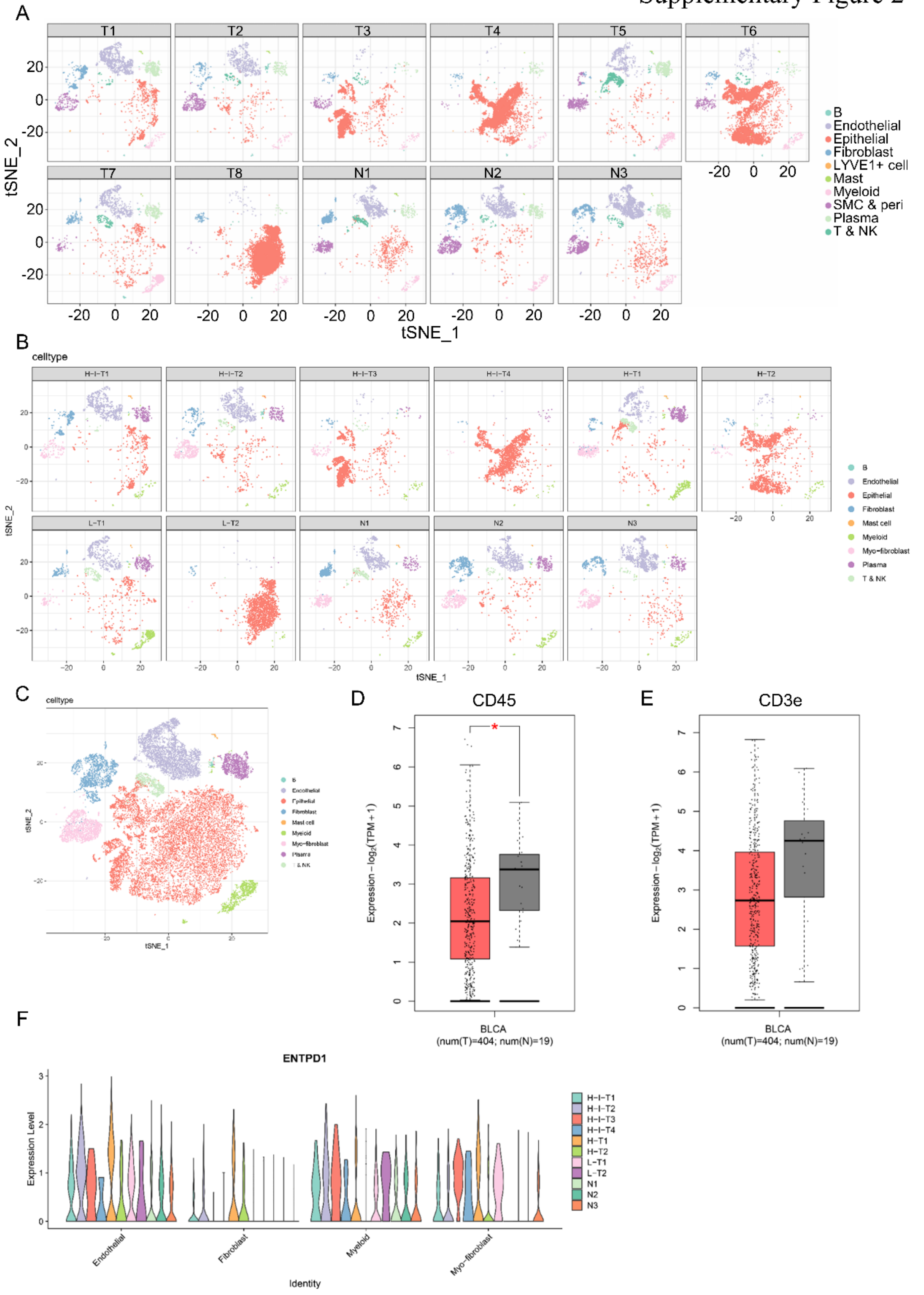

Supplementary Figure 2. Single cell cluster analysis and CD39 expression distribution in BC and paracancerous tissues. A. All the single cells from 8 BC and 3 paracancer tissues were clustered into 10 major clusters. B. After normalizing the

number of cells in each sample, 2000 single cells were randomly selected from the data of each sample (8 tumor samples and 3 paratumor samples from BC patients), and then clustered into 10 major clusters. C. The clustering diagram of 22, 000 single cells collected randomly from the 11 samples. D, E. The results of GEPIA2[3] analysis of the TCGA-BLCA data showed that there were more immune cells in the paracancerous tissues (n=19) compared with the cancer tissues (n=404). Bounds of the box spans from 25% percentile to 75% percentile, a dashed line shows median, and whiskers indicate minima and maxima. The method for differential analysis is one-way ANOVA. F. Expression and distribution of CD39 (ENTPD1) in cells from 8 tumor samples and 3 paratumor samples. P values < 0.05 were considered significant: $^{*}P < 0.05$; $^{**}P < 0.01$; $^{***}P < 0.001$; $^{****}P < 0.0001$.

Supplementary Figure 3

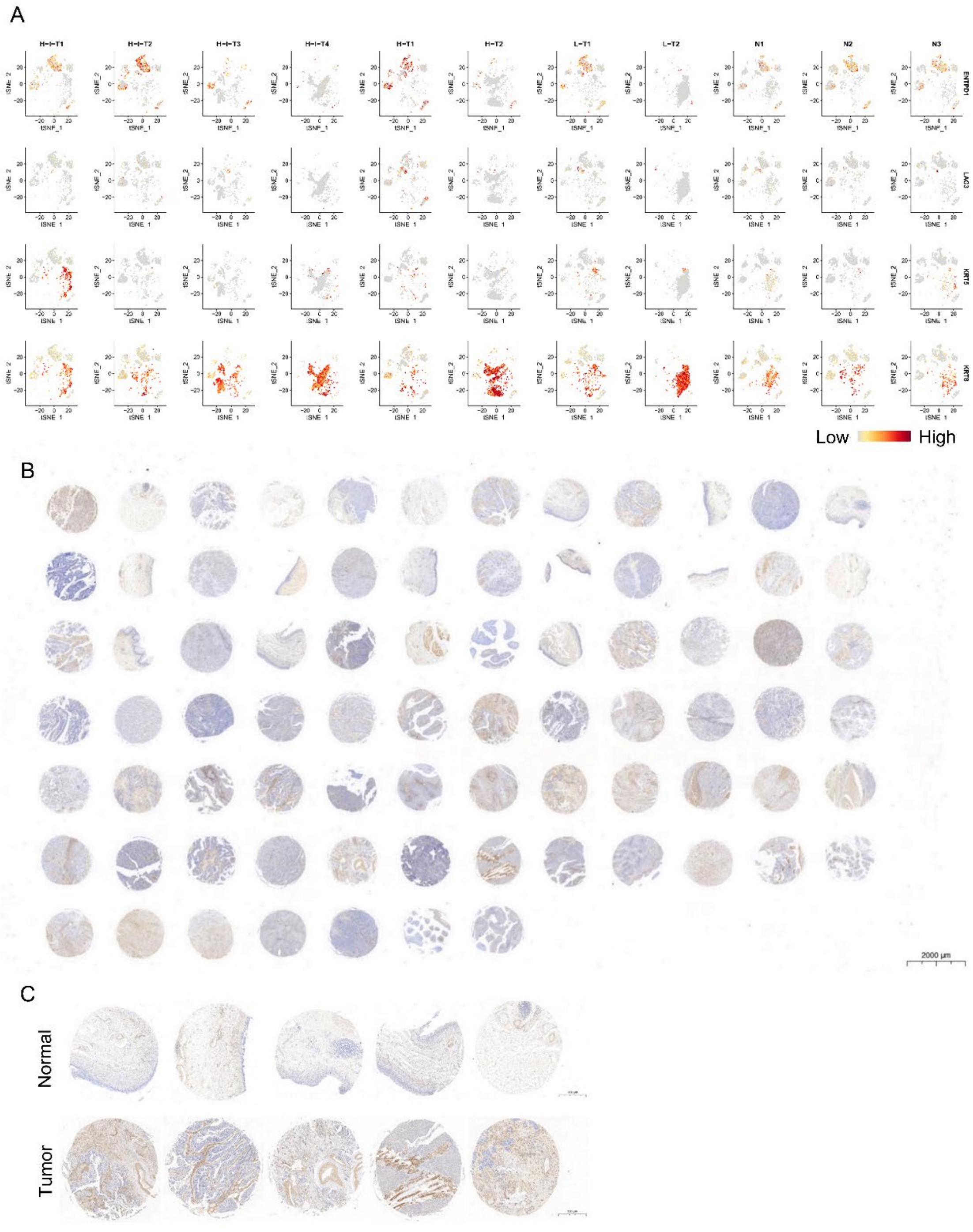

Supplementary Figure 3. Expression of CD39 in BC and paracancer tissues. A. The expression of CD39, LAG3, CK5, and CK8 in 8 BC and 3 paracancer tissues with equal cell counts. B. Gross images of immunohistochemical staining of CD39 in a tissue array including a total of 63 cancer tissues (Mean ± SEM: 5.651 ± 0.4354, n=63) and 16 cancer-adjacent normal tissues (Mean ± SEM: 2.188 ± 0.4105, n=16) from patients with BC, scale bars = 2000 µm. C. Independent cores each from 5 normal tissues and 5 cancer tissues, scale bars = 500 µm.

Supplementary Figure 4

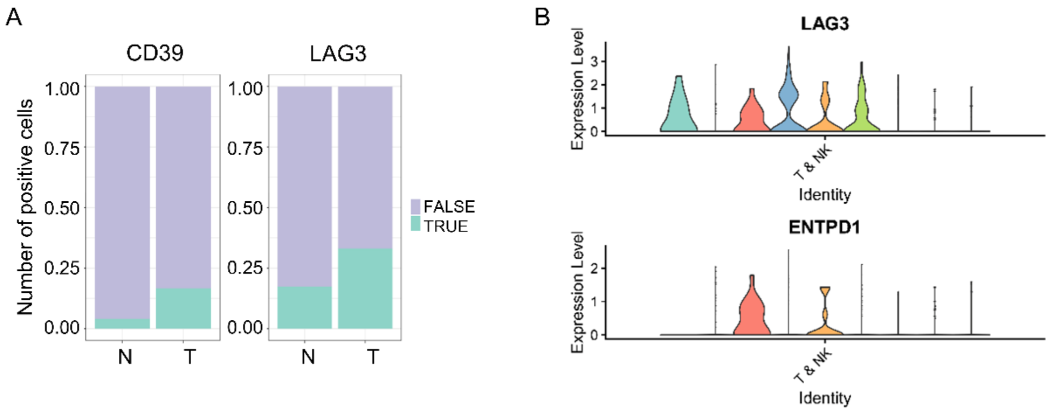

Supplementary Figure 4. Correlations between the expression levels of CD39 and LAG3 in the T & NK cells derived from BC and paracancer tissues. A. The proportion of CD39-positive lymphocytes and LAG3-positive lymphocytes derived from 8 tumor tissues was significantly higher than that of 3 normal tissues. B. A significant up-regulation of T & NK cells' surface CD39 and LAG3 expression in the tumor.

Supplementary Figure 5

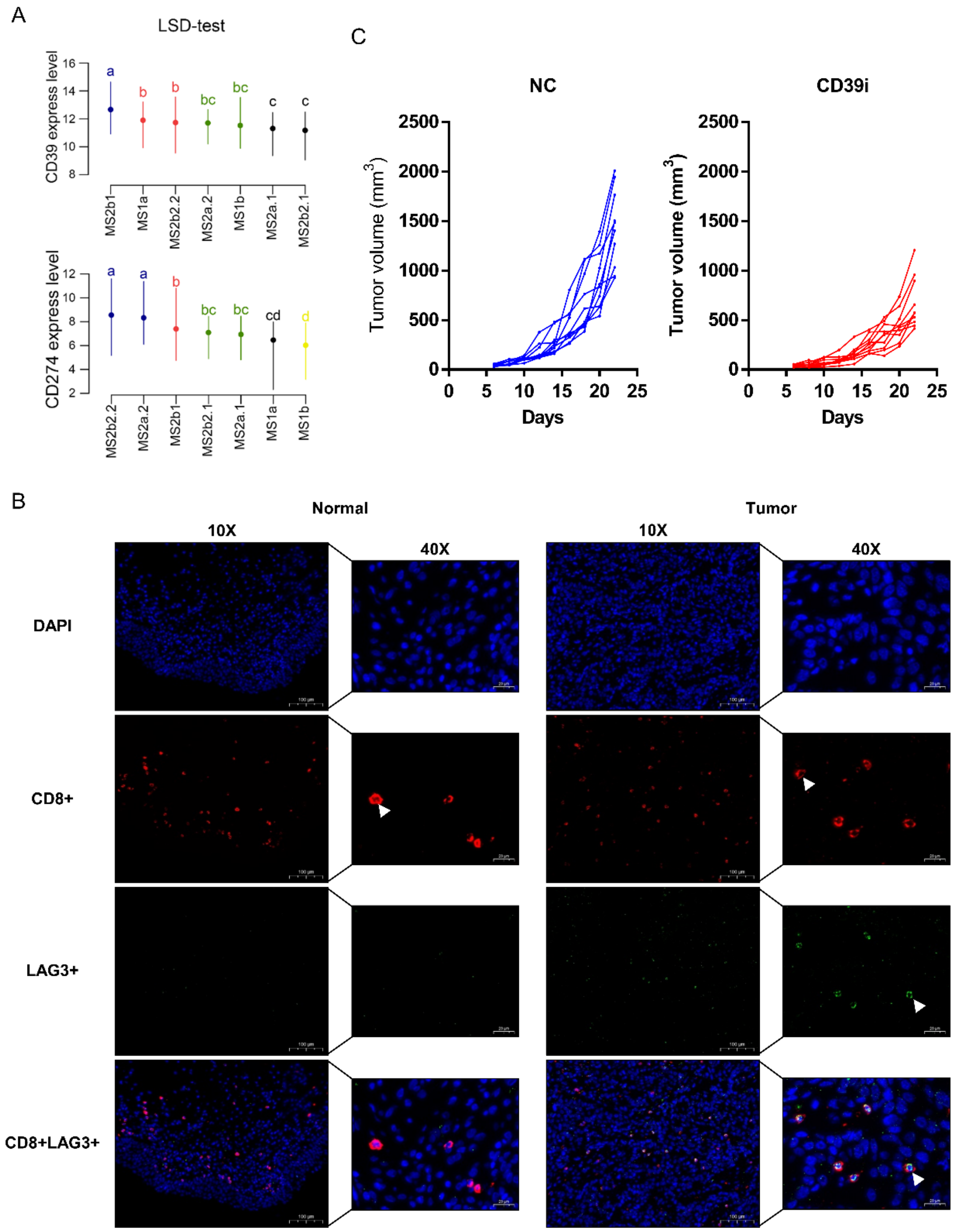

Supplementary Figure 5. Correlations between the expression levels of CD39 and PD-L1 and the proportions of T cell exhaustion in BC and paracancer tissues. A. The expression levels of PD-L1 and CD39 in 7 molecular types of BC patients reported by Mariathasan et al.[4]. Different color markers indicated that the difference was statistically significant ($P < 0.05$), MS1a: n=23, MS1b: n=79, MS2a1: n=45, MS2a2:

n=25, MS2b1: n=92, MS2b2.1: n=18, MS2b2.2: n=66. Bar graphs show the 25% percentile to 75% percentile. B. The proportion of exhausted T cells in BC tissues was remarkably greater than that in paracancer tissues (DAPI: blue, CD8: red, LAG3: green). Mean ± SEM of CD8+LAG3+ IRS: 2.778 ± 0.2971, n=63, Mean ± SEM of CD8+ IRS: 3.698 ± 0.3108, n=63. Scale bars, 100 μm (right) and 20 μm (left). C. Growth curves of individual tumors in the control group and CD39i treatment group, n=10 for each group, source data are provided as a Source Data file.

Supplementary Figure 6

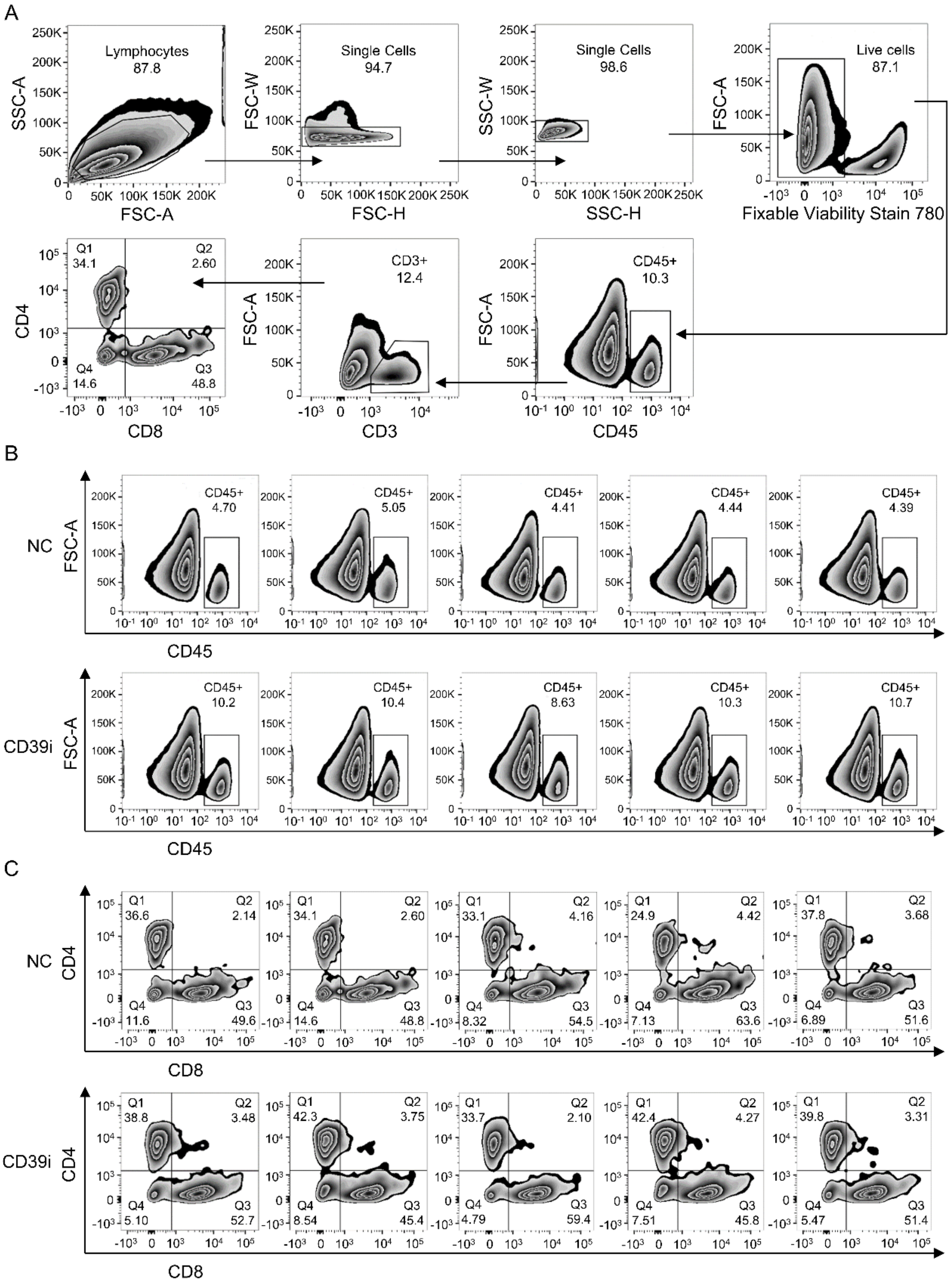

Supplementary Figure 6. CD39i treatment induced a significant increase in tumor infiltrated immune cells. A. Flow cytometry analysis strategy for T cells. Flow cytometry analysis showed that CD39i treatment induced remarkable increases in tumor infiltrated CD45+ cells (B), and CD4+ and CD8+ T cells (C). The flow cytometry analyses were repeated 3 times with 5 samples in each group.

Supplementary Figure 7

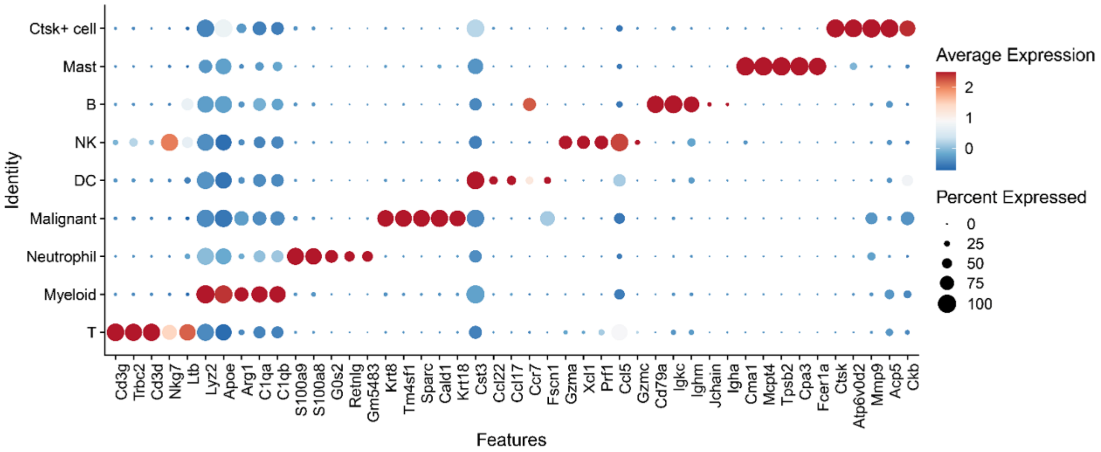

Supplementary Figure 7. Known markers used in the clustering analysis of single cells isolated from the control and CD39i treatment groups.

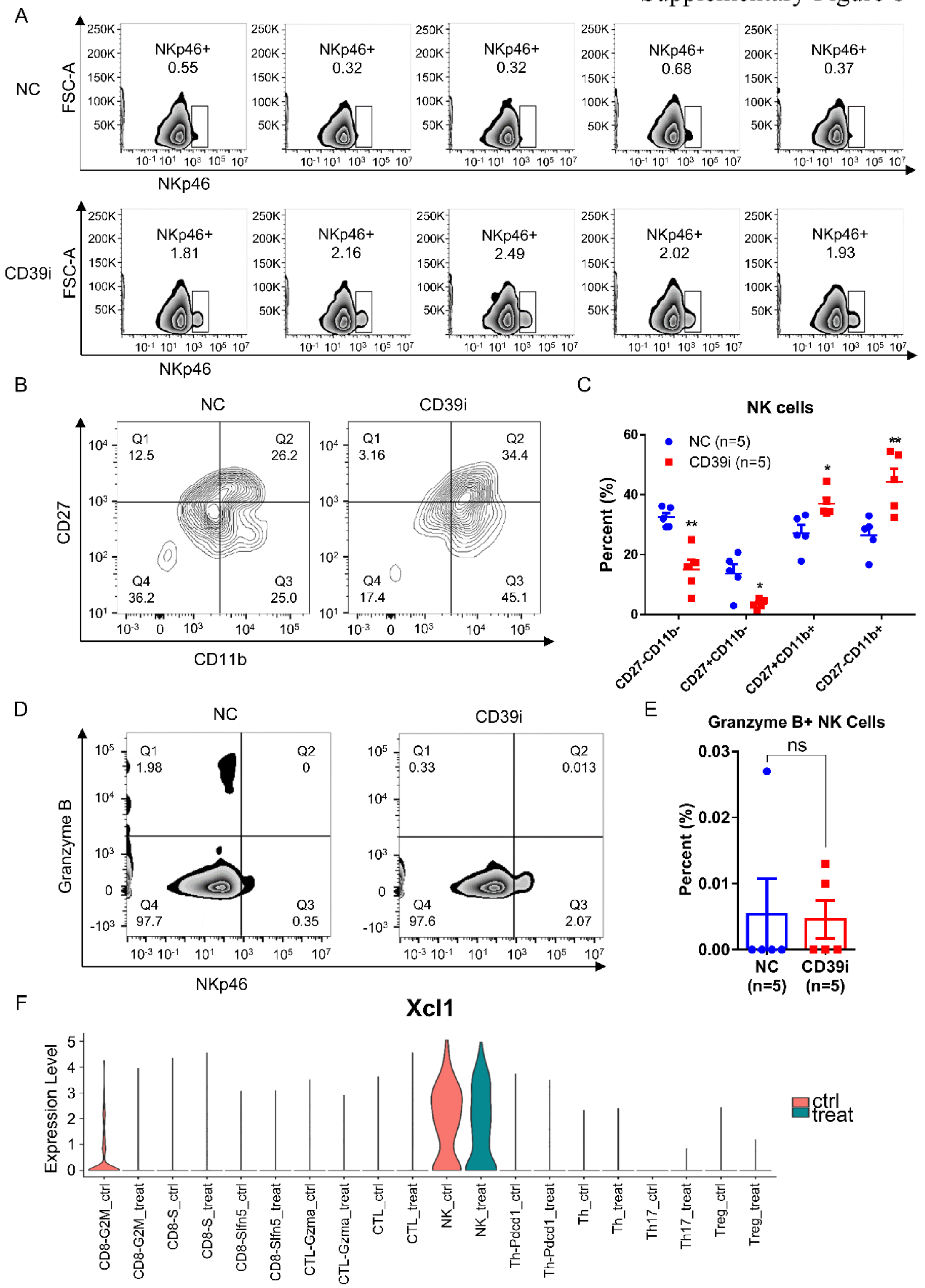

Supplementary Figure 8. CD39i treatment increased the tumor infiltrated NK cells and enhanced their function. A. Flow cytometry analysis showed that CD39i treatment increased the tumor infiltrated NK cells. B, C. CD39i treatment significantly increased the proportion of CD27+CD11b+ (Mean ± SEM: 27.18 ± 2.722 vs. 37.02 ± 1.994) and CD27-CD11b+ NK cells (Mean ± SEM: 26.54 ± 2.769 vs. 44.32 ± 4.404),

but decreased the proportion of CD27-CD11b- (Mean ± SEM: 32.50 ± 1.495 vs. 15.03 ± 3.253) and CD27+CD11b- NK cells (Mean ± SEM: 13.78 ± 3.041 vs. 3.616 ± 0.681). The two-side unpaired Student's t test was used for two-group comparisons of values. D, E. There is no difference in the proportion of Granzyme B+ NK cells between control and CD39i treated group (Mean ± SEM: 0.0054 ± 0.0054 vs. 0.0046 ± 0.0029). The two-side unpaired Student's t test was used for two-group comparisons of values. All the flow cytometry analyses were repeated 3 times with 5 samples in each group. F. CD39i treatment did not change the expression level of XCL1 on a per immune cell basis. Source data are provided as a Source Data file (C, E). P values < 0.05 were considered significant: *P < 0.05; **P < 0.01; ***P < 0.001; ****P < 0.0001.

Supplementary Figure 9

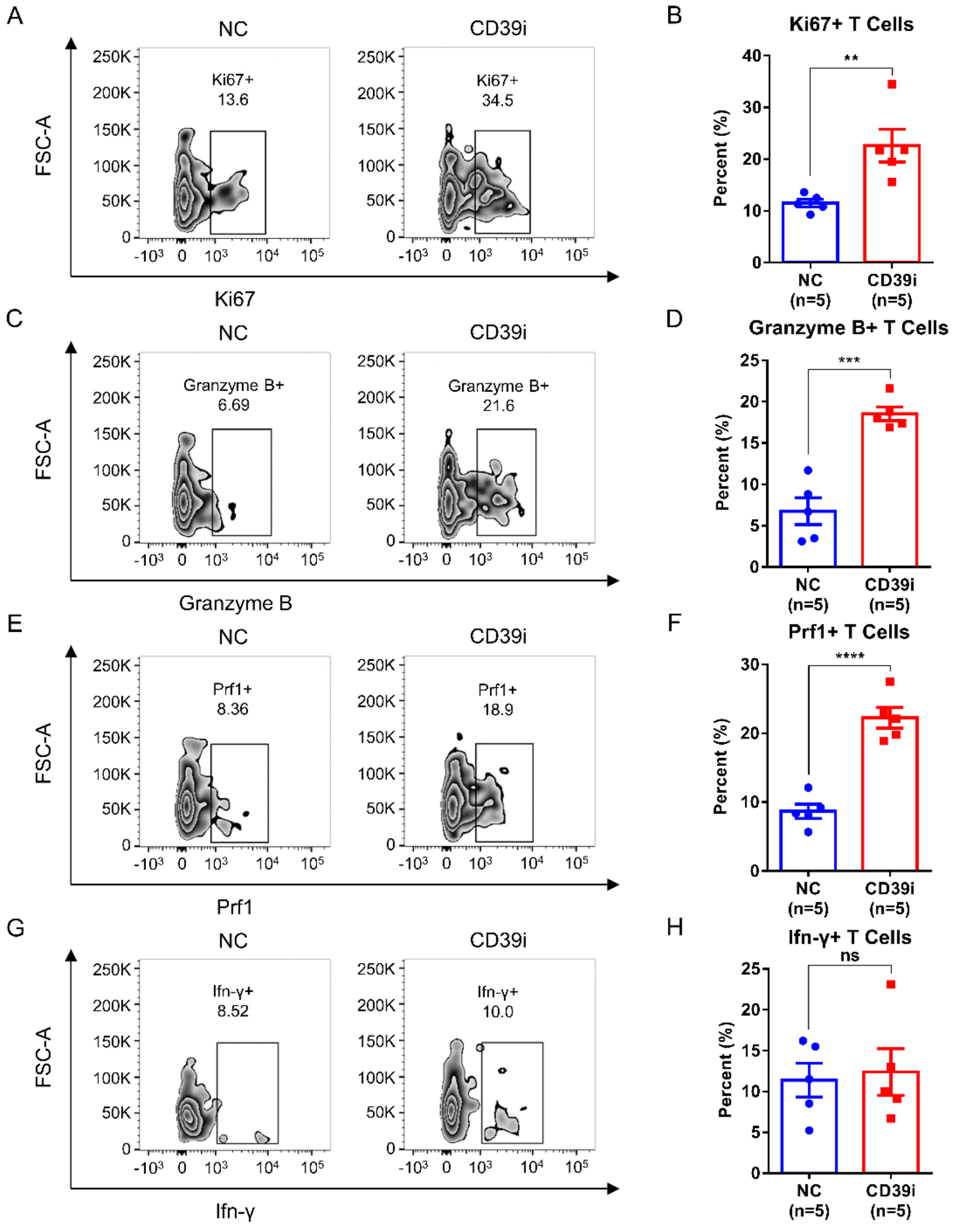

Supplementary Figure 9. The effect of CD39i on T cell function. Flow cytometry

analysis showed that CD39i treatment promoted T cell proliferation (A, B) and the secretion of granzyme B (C, D), and perforin (E, F), but not IFN-γ (G, H). Mean ± SEM: (B) 11.50 ± 0.736 vs. 22.62 ± 3.175, (D) 6.76 ± 1.623 vs. 18.54 ± 0.828, (F) 8.67 ± 1.039 vs. 22.26 ± 1.506, (H) 11.39 ± 2.078 vs. 12.38 ± 2.863. The two-side unpaired Student's t test was used for two-group comparisons of values. All the flow cytometry analyses were repeated 3 times with 5 samples in each group. Source data are provided as a Source Data file (B, D, F, H). P values < 0.05 were considered significant: *P < 0.05; **P < 0.01; ***P < 0.001; ****P < 0.0001.

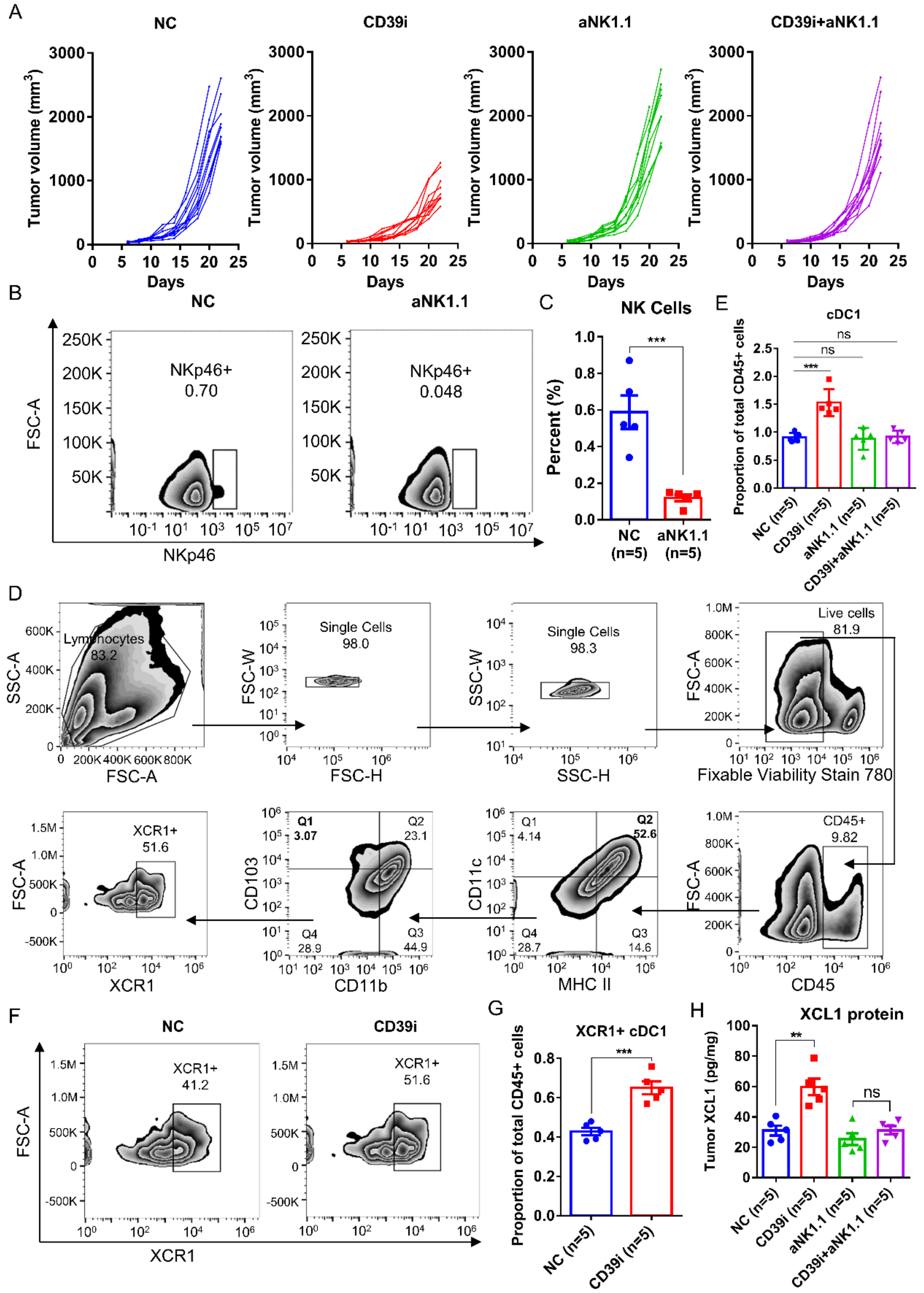

Supplementary Figure 10. Depletion of NK cells reverses the antitumor effects of CD39i in *vivo*. A. CD39i prominently inhibited the tumor growth compared with that in the control group, but the effect was remarkably reversed in the NK cells-depleted mice, n=10 for each group. B, C. Application of an aNK1.1 antibody significantly reduced NK cells within the tumor (Mean ± SEM: 0.5880 ± 0.0906 vs. 0.1196 ± 0.0186).

D. The gating strategy used to define cDC1 (CD45+CD11c+MHC II+CD103+CD11b-XCR1+). E. CD39i treatment increased the proportion of tumor infiltrated cDC1 in control mice, but not in NK cells-depleted mice. Mean ± SEM: NC (0.906 ± 0.0371), CD39i (1.530 ± 0.1081), aNK1.1 (0.878 ± 0.0878), CD39i + aNK1.1 (0.920 ± 0.0497). F, G. CD39i treatment increased the proportion (%) of XCR1+ cDC1 in tumor of WT mice (Mean ± SEM: 0.428 ± 0.0193 vs. 0.650 ± 0.0332). All the flow cytometry analyses were repeated at 3 times with 5 samples in each group. H. CD39i treatment increased tumor XCL1 protein levels in control mice (Mean ± SEM: 30.98 ± 3.24 vs. 59.68 ± 5.44), but not in NK cells absent mice (Mean ± SEM: 25.42 ± 3.86 vs. 31.14 ± 2.78), 5 samples per group, repeated 3 times. The two-side unpaired Student's t test was used for two-group comparisons of values (C, E, G, H). Source data are provided as a Source Data file (A, C, E, G, H). P values < 0.05 were considered significant: *P < 0.05; **P < 0.01; ***P < 0.001; ****P < 0.0001.

Supplementary Figure 11

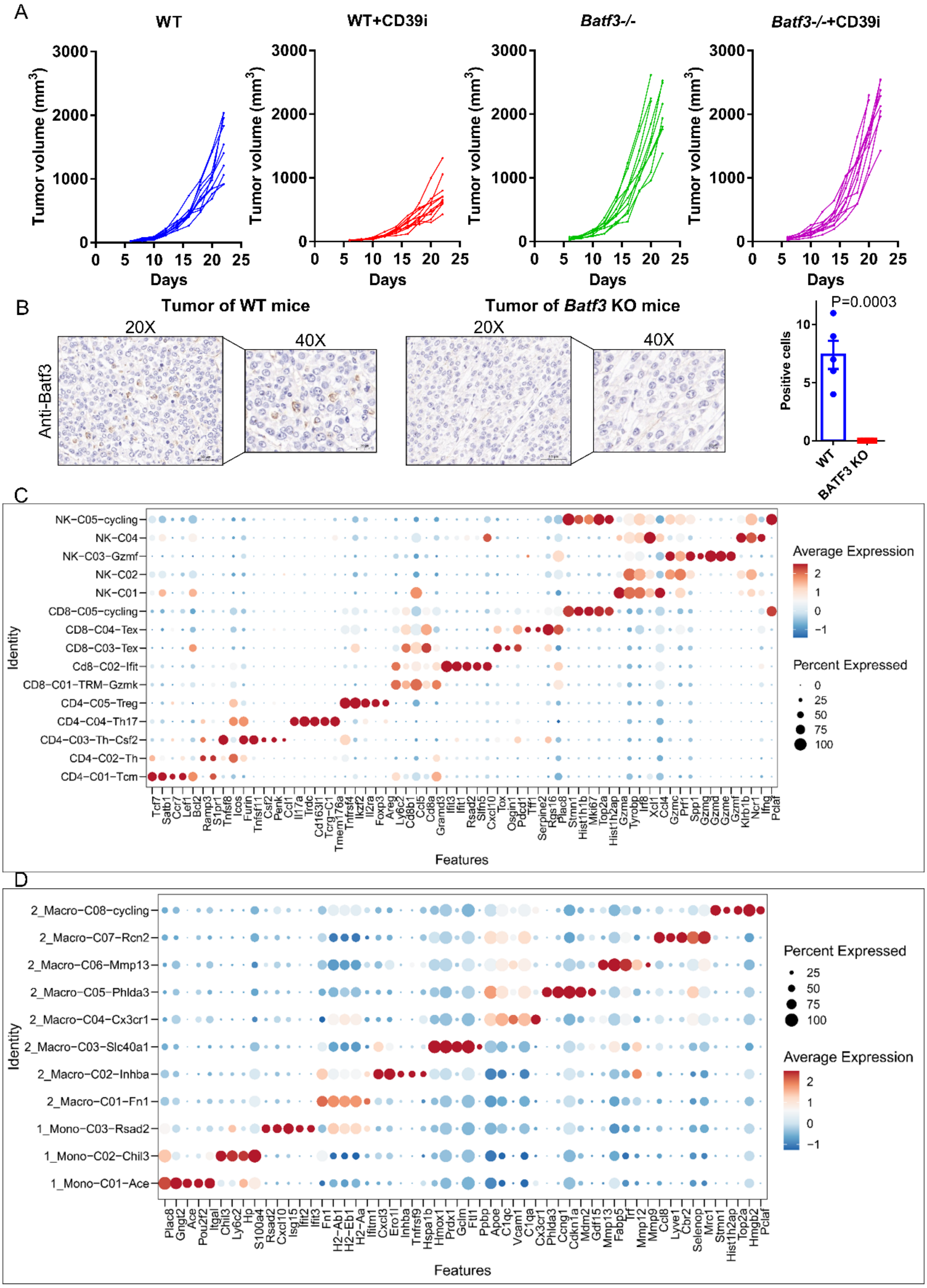

Supplementary Figure 11. cDC1-deficient leads to therapeutic failure of CD39i and the known markers for clustering analysis. A. The individual tumor growth curves of the WT, WT + CD39i, Batf3$^{-/-}$, Batf3$^{-/-}$ + CD39i groups, n=10 for each group. B. The difference in BATF3 expression between Batf3$^{+/+}$ and Batf3$^{-/-}$ cDC1-deficient mouse tumors was confirmed by IHC (Mean ± SEM: 7.400 ± 1.208 vs. 0.0 ± 0.0), 5

samples per group, repeated 3 times. Scale bars, 50 µm (right) and 20 µm (left). The two-side unpaired Student's t test was used for two-group comparisons of values. Known markers for clustering analysis of lymphocytes (C) and the monocyte-macrophage lineage (D). Source data are provided as a Source Data file (A, B).

Supplementary Figure 12

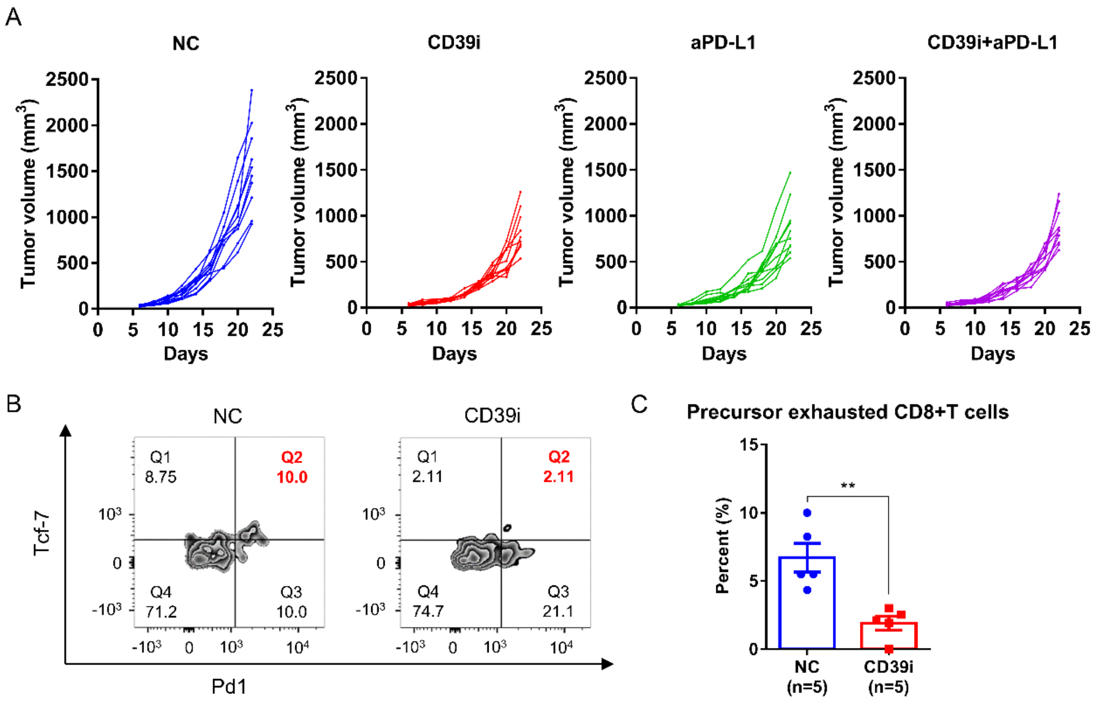

Supplementary Figure 12. Effect of the combination strategy of CD39i and aPD-L1. A. The individual tumor growth curves of the NC, CD39i, aPD-L1, and aPD-L1+CD39i groups, n=10 for each group. B, C. Flow cytometry analysis revealed that CD39i treatment significantly reduced the number of precursor exhausted T cells (CD45+CD3+CD8+PD-1+TCF-7/TCF-1+), Mean ± SEM: 6.720 ± 1.041 vs. 1.918 ± 0.515. The two-side unpaired Student's t test was used for two-group comparisons of values. The flow cytometry analyses were repeated 3 times with 5 samples in each group. Source data are provided as a Source Data file (A, C). P values < 0.05 were considered significant: *$P < 0.05$; **$P < 0.01$; ***$P < 0.001$; ****$P < 0.0001$.

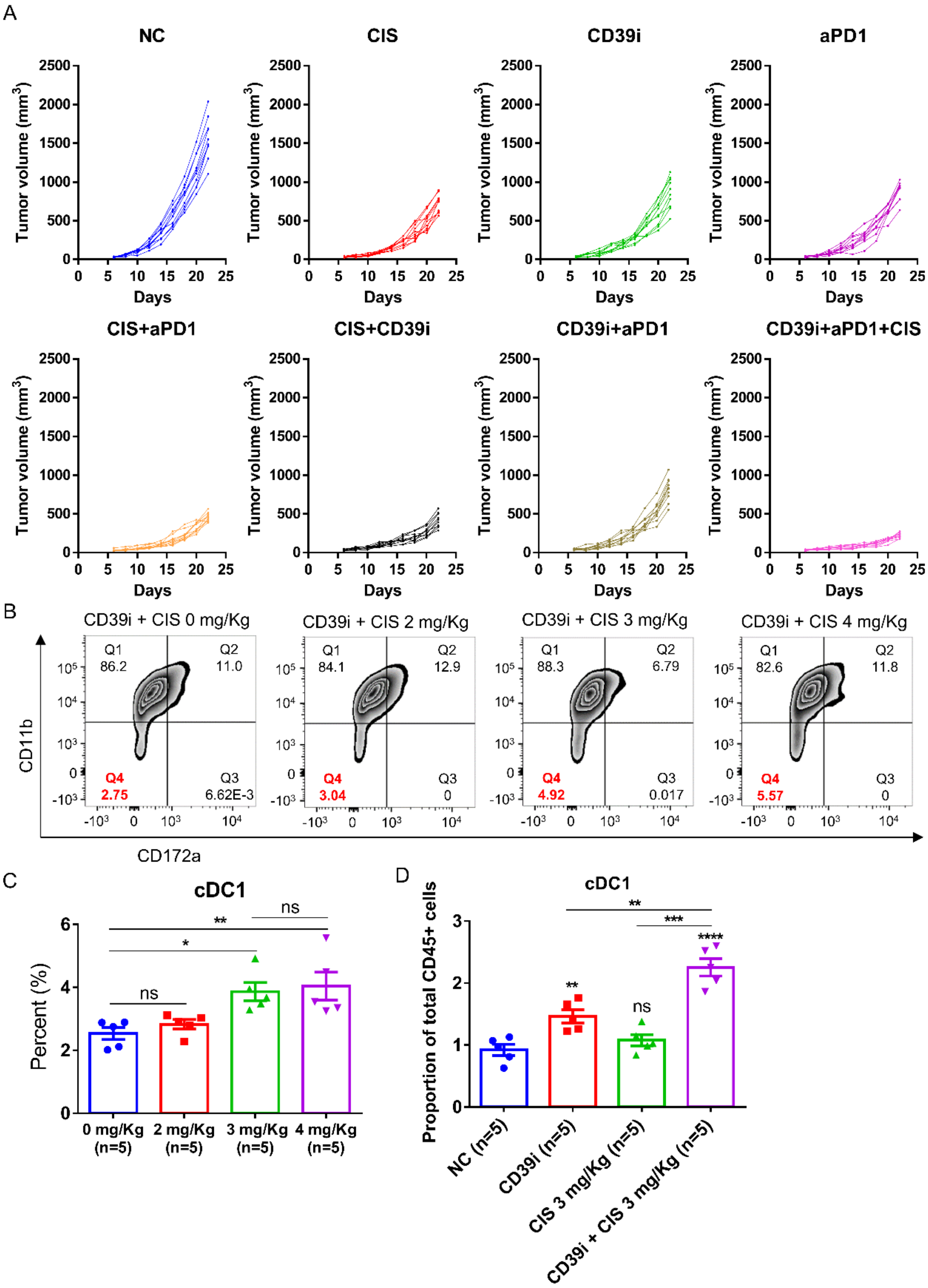

Supplementary Figure 13. Effects of different combination strategies of CD39i, aPD1 and CIS. A. The individual tumor growth curves of different combination strategies of CD39i, aPD1 and CIS, n=10 for each group. B, C. Flow cytometry analysis showed that cisplatin at doses ≥ 3mg/kg had a synergistic effect with CD39i on increasing the proportion of cDC1 (CD45+CD11c+MHC-II+CD11b-CD172a–) in

tumors. Mean ± SEM: 0 mg/kg (2.532 ± 0.1909), 2 mg/kg (2.828 ± 0.1481), 3 mg/kg (3.866 ± 0.2851), 4 mg/kg (4.040 ± 0.4412). D. CD39i (but not 3mg/kg cisplatin alone) treatment significantly increased the proportion of tumor infiltrated cDC1 cells (cDC1: CD45+CD11c+MHC II+CD103+CD11b-), and cisplatin at dose of 3mg/kg had a synergistic effect with CD39i on increasing the proportion of cDC1 in tumors. Mean ± SEM: NC (0.9220 ± 0.0909), CD39i (1.464 ± 0.1070), CIS 3 mg/kg (1.078 ± 0.0903), CD39i + CIS 3 mg/kg (2.254 ± 0.1399). The two-side unpaired Student's t test was used for two-group comparisons of values (C, D). All the flow cytometry analyses were repeated 3 times with 5 samples in each group. Source data are provided as a Source Data file (A, C, D). P values < 0.05 were considered significant: *$P < 0.05$; **$P < 0.01$; ***$P < 0.001$; ****$P < 0.0001$.

Supplementary References

1. Chen Z*, et al.* Single-cell RNA sequencing highlights the role of inflammatory cancer-associated fibroblasts in bladder urothelial carcinoma. *Nature communications* **11**, 5077 (2020).
2. Tang Z, Li C, Kang B, Gao G, Li C, Zhang Z. GEPIA: a web server for cancer and normal gene expression profiling and interactive analyses. *Nucleic acids research* **45**, W98-w102 (2017).
3. Tang Z, Kang B, Li C, Chen T, Zhang Z. GEPIA2: an enhanced web server for large-scale expression profiling and interactive analysis. *Nucleic acids research* **47**, W556-w560 (2019).
4. Mariathasan S*, et al.* TGFβ attenuates tumour response to PD-L1 blockade by contributing to exclusion of T cells. *Nature* **554**, 544-548 (2018).
