# Supplementary material for: Single cell sequencing reveals that CD39 inhibition mediates changes to the tumor microenvironment
Source: Nat Commun. 2022 Nov 8;13:6740. doi: 10.1038/s41467-022-34495-z (PMC9643495; doi:10.1038/s41467-022-34495-z)
Supplement: Supplementary file 2 — Reporting Summary [file 41467_2022_34495_MOESM2_ESM.pdf]

nature portfolio

Corresponding author(s): Ke Chen

Last updated by author(s): Sep 6, 2022

Reporting Summary

Nature Portfolio wishes to improve the reproducibility of the work that we publish. This form provides structure for consistency and transparency in reporting. For further information on Nature Portfolio policies, see our Editorial Policies and the Editorial Policy Checklist.

Statistics

For all statistical analyses, confirm that the following items are present in the figure legend, table legend, main text, or Methods section.

n/a	Confirmed	
☐	☒	The exact sample size (*n*) for each experimental group/condition, given as a discrete number and unit of measurement
☐	☒	A statement on whether measurements were taken from distinct samples or whether the same sample was measured repeatedly
☐	☒	The statistical test(s) used AND whether they are one- or two-sided<br>*Only common tests should be described solely by name; describe more complex techniques in the Methods section.*
☒	☐	A description of all covariates tested
☐	☒	A description of any assumptions or corrections, such as tests of normality and adjustment for multiple comparisons
☐	☒	A full description of the statistical parameters including central tendency (e.g. means) or other basic estimates (e.g. regression coefficient) AND variation (e.g. standard deviation) or associated estimates of uncertainty (e.g. confidence intervals)
☐	☒	For null hypothesis testing, the test statistic (e.g. *F*, *t*, *r*) with confidence intervals, effect sizes, degrees of freedom and *P* value noted<br>*Give P values as exact values whenever suitable.*
☒	☐	For Bayesian analysis, information on the choice of priors and Markov chain Monte Carlo settings
☒	☐	For hierarchical and complex designs, identification of the appropriate level for tests and full reporting of outcomes
☐	☒	Estimates of effect sizes (e.g. Cohen's *d*, Pearson's *r*), indicating how they were calculated

Our web collection on statistics for biologists contains articles on many of the points above.

Software and code

Policy information about availability of computer code

Data collection	There was no software used for data collection.
Data analysis	Flow cytometry was performed using a flow cytometer (BD LSRFortessa X-20, USA), and analysis was performed using FlowJo: Flow Cytometry Analysis Software, v10.0.7 (Tree Star).<br>Single cell RNA-sequencing was performed as described in our previous work (PMID: 33033240). In brief, subcutaneous tumors from 3 mice in each group were dissociated to obtain single cell suspensions of immune cells, which were then mixed 1:1:1 and were loaded on a 10X Chromium Controller (10X Genomics) to generate nanoliter-scale gel beads-in-emulsions (GEMs). Barcoded scRNA-seq libraries were prepared using the Chromium Single Cell 3' v3 Reagent Kit. Then, the GEMs were used to generate barcoded, full-length cDNA through reverse transcription reactions. Next, the barcoded, full-length cDNA was used for library construction via fragmentation, end repair, A-tailing, ligation to an index adaptor, and amplification by PCR. The final libraries were sequenced on a HiSeq X Ten platform (Illumina), and 150 bp paired-end reads were generated.<br>Cell Ranger (version 5.0.0) was used to process the raw data and generate the UMI matrix. Cells with fewer than 1000 UMIs, or with over 20% percent of transcripts derived from mitochondria were considered as low-quality cells and were discarded. All the downstream analyses were performed with Seurat (v 3.0.1) in the R environment (version 3.6.1). Considering that a library of each sample was constructed respectively, sample IDs were used to remove potential batch effects with Harmony (https://github.com/immunogenomics/harmony). After principal component analysis (PCA) analysis, the top 50 PCs were used to perform tSNE or UMAP analysis. Clusters found by the FindClusters function of Seurat were annotated with known markers as listed in the Results. Heatmaps of selected genes were visualized with scanpy (version 1.7.1).<br>The prepared sections were scanned as high-resolution digital images at 5.4× using a Pannoramic MIDI II scanner (3DHISTECH Ltd., Budapest, Hungary).<br>All analyses were finished using the GraphPad Prism 6.02 software (GraphPad Software Inc., San Diego, CA, USA).

For manuscripts utilizing custom algorithms or software that are central to the research but not yet described in published literature, software must be made available to editors and reviewers. We strongly encourage code deposition in a community repository (e.g. GitHub). See the Nature Portfolio guidelines for submitting code & software for further information.

Data

Policy information about availability of data

All manuscripts must include a data availability statement. This statement should provide the following information, where applicable:

- Accession codes, unique identifiers, or web links for publicly available datasets
- A description of any restrictions on data availability
- For clinical datasets or third party data, please ensure that the statement adheres to our policy

The TCGA-BLCA publicly available data used in this study are available in the UCSC XENA database under accession code http://xena.ucsc.edu/. The single-cell RNA sequencing data of the 8 bladder cancer and 3 para-cancer tissues of bladder cancer patients generated in this study are available in GSA-Human (https://ngdc.cncb.ac.cn/gsa-human/) under the accession code HRA000212 and in SRA datasets (https://www.ncbi.nlm.nih.gov/sra) under BioProject PRJNA662018. The single-cell RNA sequencing data of mice in different treatment groups (untreated, CD39i, WT-untreated, WT-CD39i, Batf3-/--untreated, and Batf3-/--CD39i) are deposited in the Gene Expression Omnibus (GEO) database (https://www.ncbi.nlm.nih.gov/geo/) under accession number GSE189127. The IMvigor210 dataset21 was exported from the R package IMvigor210CoreBiologies under accession code http://research-pub.gene.com/IMvigor210CoreBiologies/. The remaining data are available within the Article, Supplementary Information or Source Data file provided with this paper.

Field-specific reporting

Please select the one below that is the best fit for your research. If you are not sure, read the appropriate sections before making your selection.

- [x] Life sciences
- [ ] Behavioural & social sciences
- [ ] Ecological, evolutionary & environmental sciences

For a reference copy of the document with all sections, see nature.com/documents/nr-reporting-summary-flat.pdf

Life sciences study design

All studies must disclose on these points even when the disclosure is negative.

Sample size	We determined the number of mice per group to be 10 according to the request of the Animal Care and Use Committee of Tongji Medical College of Huazhong University of Science and Technology and the numerous reports of animal studies.<br>Subcutaneous tumors of 3 mice in each group (Control, CD39i, Batf3+/+, Batf3−/−) were dissociated to obtain single cell suspensions of immune cells, which were then mixed 1:1:1 and profiled at single cell level.<br>The immunofluorescence and immunohistochemistry staining assay were performed on a tissue chip including a total of 63 cancer tissues and 16 cancer-adjacent normal tissues from patients with bladder cancer.
Data exclusions	No data were excluded from analysis.
Replication	All results represent the mean±S.E.M. from three independent experiments. All the replication were successful.
Randomization	After the construction of the mouse tumor model, the mice were randomly divided into different groups according to the random number table for subsequent treatment. After drug intervention, a certain number of mice were randomly selected from different groups according to the random number table for subsequent analysis such as flow cytometry and single-cell sequencing.
Blinding	Blinding was not included in the study, as this study did not involve clinical trials/clinical trial associated data.

Reporting for specific materials, systems and methods

We require information from authors about some types of materials, experimental systems and methods used in many studies. Here, indicate whether each material, system or method listed is relevant to your study. If you are not sure if a list item applies to your research, read the appropriate section before selecting a response.

Materials & experimental systems

n/a	Involved in the study	
☐	☒	Antibodies
☐	☒	Eukaryotic cell lines
☒	☐	Palaeontology and archaeology
☐	☒	Animals and other organisms
☒	☐	Human research participants
☒	☐	Clinical data
☒	☐	Dual use research of concern

Methods

n/a	Involved in the study	
☒	☐	ChIP-seq
☐	☒	Flow cytometry
☒	☐	MRI-based neuroimaging

Antibodies

Antibodies used	Bio X cell: monoclonal anti-mouse NK1.1 antibody (cat #: BE0036, Bio X cell, NH, USA), monoclonal anti-mouse PD-1 (CD279) antibody (cat #: BE0146, Bio X cell, NH, USA), monoclonal anti-mouse PD-L1 (B7-H1) antibody (cat #: BE0101, Bio X cell, NH, USA).<br>BD (San Diego, USA): anti-mouse CD45-FITC (1: 100, cat #: 553079), anti-mouse CD4-BB700 (1: 100, cat #: 566407), anti-mouse CD8a-PE-Cyanine7 (1: 100, cat #: 552877), anti-mouse CD3e-BV510 (1: 50, cat #: 563024), anti-mouse CD335/NKp46-Alexa 647 (1: 100, cat #:560755), anti-mouse IFN-γ-BV421 (1: 50, cat #: 563376), anti-mouse Ki-67-BV605 (1: 50, cat #: 567122), anti-mouse TCF-7/TCF-1-Alexa 647 (1: 50, cat #: 566693), anti-mouse CD279 (PD-1)-APC-R700 (1: 50, cat #: 565815), anti-mouse CD11b-PE-Cyanine7 (1: 50, cat #: 552850), anti-mouse I-A/I-E (MHC-II)-BB700 (1: 50, cat #: 746086), anti-mouse CD11c-BV605 (1: 50, cat #: 563057), and anti-mouse CD172a/SIRPα-PE (1: 50, cat #: 560107).<br>BioLegend (San Diego, USA): anti-mouse Perforin-PE (1: 50, cat #: 154306), anti-mouse Granzyme B Recombinant-PE/Dazzle™ 594 (1: 50, cat #: 372216), anti-mouse CD27-PE (1: 50, cat #: 124210), anti-mouse CD103-APC (1: 50, cat #: 121413), and anti-mouse/rat XCR1-BV421 (1: 50, cat #: 148216).<br>The cell viability was monitored using Fixable Viability Stain 780 (1: 100, APC-Cyanine7, cat #: 565388, BD HorizonTM).<br>The anti-CD39 (1:1000, Abcam, ab223842), anti-BATF3 (1:100, Abbkine, ABP57435) and HRP anti-Rabbit IgG antibody (1: 200, Servicebio, G1213) used in IHC.<br>The anti-CD8 (1:100, Abcam, ab178089) and anti-LAG3 (1:1000, Abcam, ab209236) were used for IFC.
Validation	The validation of all of the antibodies depends on product datasheet and published literature.<br>Anti-CD39 (Reacts with: Mouse, Human, 1:1000, Abcam, ab223842), Anti-BATF3 (Reacts with: Mouse, Rat, Human, 1:100, Abbkine, ABP57435), Anti-CD8 (Reacts with: Human, 1:100, Abcam, ab178089), Anti-LAG3 (Reacts with: Human, 1:1000, Abcam, ab209236).<br>Bio X cell: monoclonal anti-mouse NK1.1 antibody (cat #: BE0036, Bio X cell, NH, USA), monoclonal anti-mouse PD-1 (CD279) antibody (cat #: BE0146, Bio X cell, NH, USA), monoclonal anti-mouse PD-L1 (B7-H1) antibody (cat #: BE0101, Bio X cell, NH, USA).

Eukaryotic cell lines

Policy information about cell lines

Cell line source(s)	The mouse-derived BC cell line MB49 obtained from Otwo Biotech Inc. (Guangzhou, China).
Authentication	NO.
Mycoplasma contamination	Mycoplasma contamination were negative based on the PCR results.
Commonly misidentified lines<br>(See ICLAC register)	NO.

Animals and other organisms

Policy information about studies involving animals; ARRIVE guidelines recommended for reporting animal research

Laboratory animals	Wild type (WT) C57BL/6J male mice (6- to 8-week-old) purchased from GemPharmatech Co., Ltd., Nanjing, China and Batf3-/- male mice (6- to 8-week-old) on a C57BL/6 mouse background purchased from the Animal Model Research Center of Nanjing University, Nanjing, China were housed in a pathogen-free facility at 22°C with 50% humidity and 12 h light/12 h dark cycles.
Wild animals	We did not use any wild animals.
Field-collected samples	No field collected samples were used in the study.
Ethics oversight	All animal procedures have been approved by the Animal Care and Use Committee of Tongji Medical College of Huazhong University of Science and Technology (IACUC Number: 2580).

Note that full information on the approval of the study protocol must also be provided in the manuscript.

Flow Cytometry

Plots

Confirm that:

☒ The axis labels state the marker and fluorochrome used (e.g. CD4-FITC).

☒ The axis scales are clearly visible. Include numbers along axes only for bottom left plot of group (a 'group' is an analysis of identical markers).

☒ All plots are contour plots with outliers or pseudocolor plots.

☒ A numerical value for number of cells or percentage (with statistics) is provided.

Methodology

Sample preparation	Subcutaneous tumors were collected and cut into small pieces (< 2mm in diameter), then digested with 10ml of dissociation solution (RPMI 1640 medium containing 10% FBS, 1mg/mL collagenase type IV (Biosharp, Hefei, China), 100 μg/mL DNase I

	(Biosharp, Hefei, China), and 2.5 μg/mL hyaluronidase (Biosharp, Hefei, China) for 60 min on a 37°C shaker. Next, 4ml RPMI 1640 medium containing 10% FBS was added to dilute the suspensions, then cell suspensions were passed through 70 μm cell strainers, and the lower layer was collected after centrifuging at 5,000 revolutions per minute for 5 min. Subsequently, the erythrocytes were lysed by red blood cell lysing buffer (Biosharp, Hefei, China) according to manufacturer's instructions, then washed and resuspended the remaining single cells in PBS.
Instrument	The flow cytometry was done using flow cytometer (BD LSRFortessa X-20, USA).
Software	FlowJo: Flow Cytometry Analysis Software, v10.0.7 (Tree Star).
Cell population abundance	The CD45+ cells occupied 10.05 ± 0.36% of whole cell population in CD39i treatment group, and 4.60 ±0.13% in Control group.
Gating strategy	NK cells were defined as CD45+CD3-CD335+, CD8+ T cells were defined as CD45+CD3+CD8+, CD4+ T cells were defined as CD45+CD3+CD4, precursor exhausted T cells (CD45+CD3+CD8+PD-1+TCF-7/TCF-1+), cDC1 (CD45+CD11c+MHC II+CD103 +CD11b-//XCR1+) or cDC1 (CD45+CD11c+MHC-II+CD11b-CD172a–)

☒ Tick this box to confirm that a figure exemplifying the gating strategy is provided in the Supplementary Information.
